# Stem Cell Applications in Human Hair Growth: A Literature Review

**DOI:** 10.7759/cureus.37439

**Published:** 2023-04-11

**Authors:** Arjavon T Talebzadeh, Nojan Talebzadeh

**Affiliations:** 1 Surgery, California Northstate University College of Medicine, Sacramento, USA; 2 Surgery, South County Surgery Center, San Diego, USA

**Keywords:** alopecia areata, androgenic alopecia, male pattern baldness, hair follicle, stem cell therapy, alopecia, stem cell, hair loss

## Abstract

Stem cells are being investigated in applications in male pattern baldness and other forms of alopecia of the human scalp. This report explores the literature regarding the various applications of stem cells and their potential for future use in the correction of multifactorial etiologies for male or female pattern baldness. Different contemporary studies revealed that stem cells may be directly injected into the scalp to allow the growth of new hair follicles in males or females for the correction of alopecia. Stem cells may also be used in growth factor stimulation of existing inactive and atrophic follicles to yet again become viable and active follicles. Additional studies indicate that various regulatory mechanisms may be used to reinitiate the existing inactive follicle cells to regrow hair in male pattern baldness. Stem cells injected into the scalp could aid these regulatory mechanisms. In the future, stem cell treatment may serve as a viable option superior to the US Food and Drug Administration (FDA)-approved invasive and noninvasive techniques currently used to combat alopecia.

## Introduction and background

**Figure 1:**
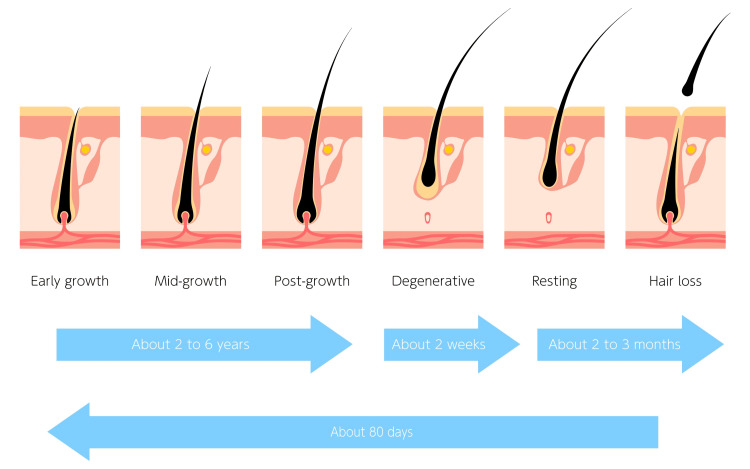
The progression and time line of hair cycle The image has been reprinted (adapted) with permission from iStockphoto (2023)

The science of hair loss is being investigated for decades. It is understood that up to 100 hair loss a day is perfectly normal [1]. Any loss above this number with no replenishment however leads to eventual thinning of the hair. Male and female hair loss has multiple etiologies. Stressors such as bereavement leading to hair loss could lead to telogen effluvium[2]. Studies have shown that under stressful conditions, cortisol secretion increases in humans. The presence of cortisol has been shown to decrease the activity of follicle stem cells located at the bottom of the hair follicle in a niche called bulge. This bulge location is positioned between the arrector pili muscle and the opening of the sebaceous gland, therefore reducing the regeneration ability of the hair [3]. The dermal papilla production of a molecular signal growth arrest-specific 6 (Gas6) is reduced. Gas6 reduction decreases the activation of stem cells leading to the loss of regeneration capability [4]. It has also been shown that the smooth muscle surrounding the hair shaft can also have a contribution to hair regression [5]. The contracture of the muscle affects the catagen phase of the hair growth cycle leading to follicle regression [6]. The interruption of cell signals in this mechanism may help reduce this regression (Figure 1).

Medications such as some beta-blockers and antidepressant and chemotherapy could have secondary hair loss known as telogen effluvium as side effects [7]. Illness such as hypothyroidism has also been associated with thinning hair, and certain dietary deficiency that may include zinc, iron, and biotin could also have same results [8]. Autoimmune conditions such as alopecia areata (AA) have also been shown to lead to hair loss. In patients with androgenic alopecia, hair loss has been associated with increased number of androgen receptors and possibly an increase the enzyme 5 alpha-reductase. This leads to the increased production of the hormone dihydrotestosterone (DHT) around hair follicles. In androgenic alopecia, the hormone DHT increases in balding scalp, and the activity of enzymes such as 5 alpha-reductase increases. Also, the number of receptors for DHT increases. It has also been shown that DHT binds to susceptible hair follicles and activates genes that increase the miniaturization of hair follicle [9,10].

**Figure 2:**
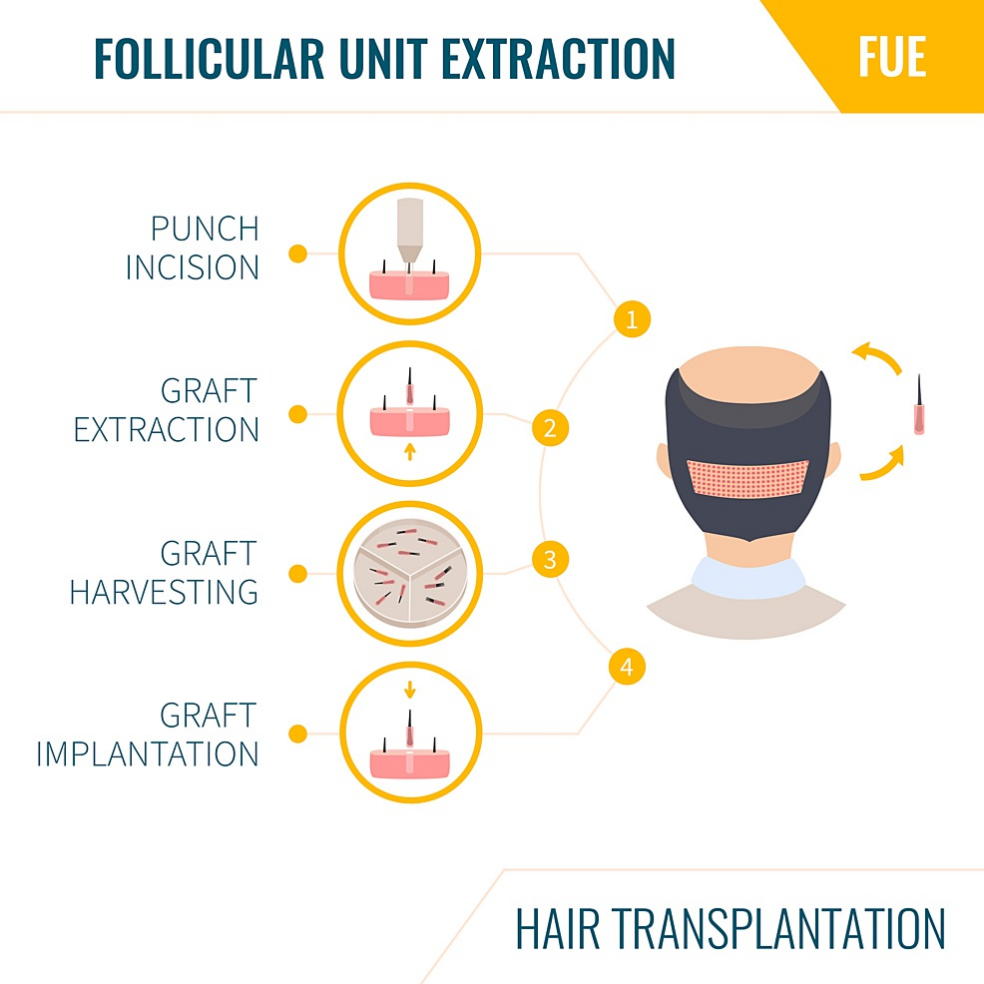
Follicular unit transplant procedure The image has been reprinted (adapted) with permission from iStockphoto (2023)

The International Society of Hair Restoration Surgery reports that about 35 million American males and 21 million American females suffer from hair loss [11]. The demand for more effective and cheaper treatment for alopecia stems from a desire by many balding individuals to maintain their hair and a significant potential for helping these patients [12]. Invasive treatment such as the use of hair plugs/transplant has been employed for decades, and such treatment has been criticized for its unnatural appearance (Figure 2).

Over-the-counter pharmaceutical drugs such as minoxidil and Propecia are viable treatment plans only when dosages are taken on a continual basis [13]. Minoxidil applied directly to scalp skin leads to vasodilation, increasing nutrients and oxygen delivery to the follicles. Finasteride acts by being the inhibitor of 5 alpha-reductase, therefore reducing the DHT levels around the follicles. This drug can only be used in males due to teratogenic side effects in pregnancy. Spironolactone, a less potent inhibitor, has been tried in females, but the side effects such as hyperkalemia, headache, breast tenderness, and fatigue have to be considered [14]. A pause in the use of such drugs would immediately lead to the continuation of hair loss at a rate that matches that observed prior to the use of the drugs.

Stem cells are now being investigated to see if the hair can be replenished using patients’ own mesenchymal cells from the base of the existing follicles, fat cells, and bone marrow stem cells or using embryonic umbilical stem cells to stimulate hair growth or replacement. Other modalities such as stem cell-derived nutrient medium and stem cell-derived exosomes are also novel approaches to future hair loss therapy.

## Review

**Figure 3:**
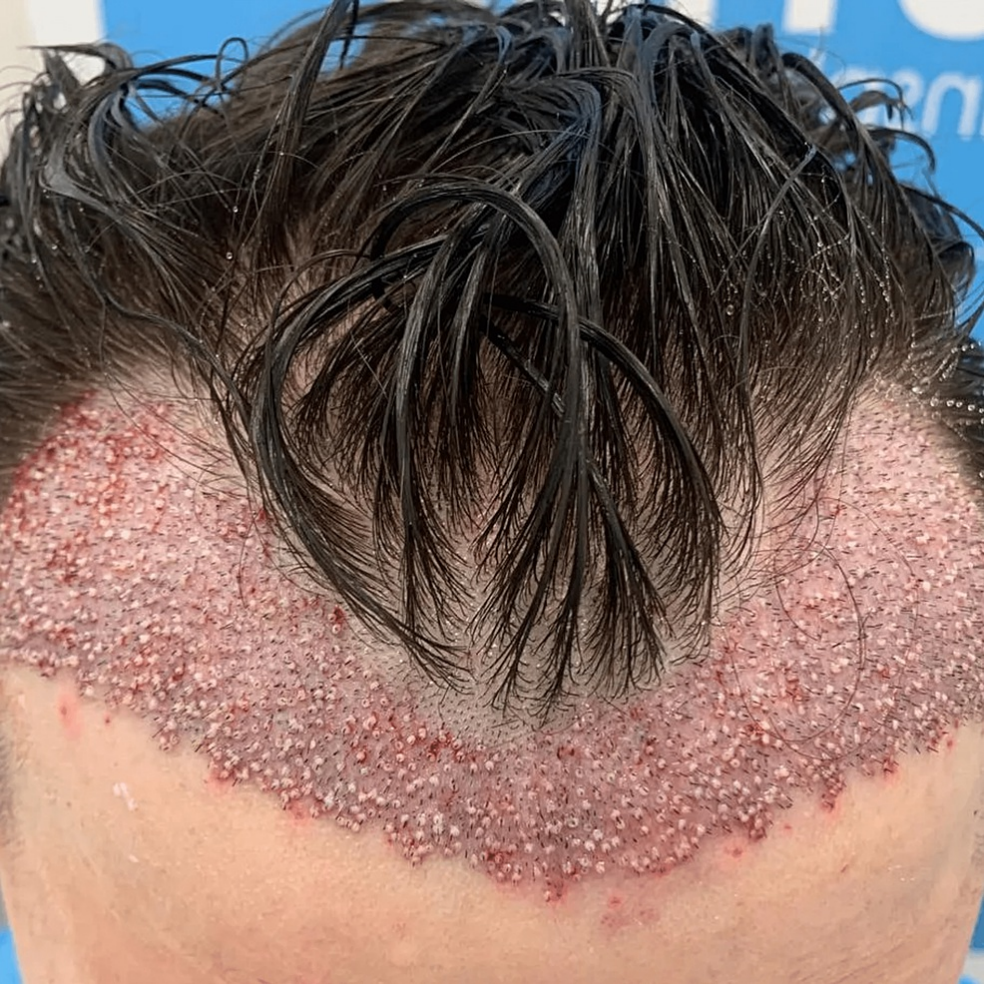
Hair transplant immediate postoperative result. This allows to establish a fuller and more anterior hairline in the patients The image has been reprinted (adapted) with permission from iStockphoto (2023)

The use of single follicular hair transplants is among the newest methods for the correction of male pattern baldness today. In this technique, donor hair can be obtained in single, double, or triple hair combinations from the posterior scalp and reinserted into the area of hair loss one follicle at a time (Figure 3) [15].

**Figure 4:**
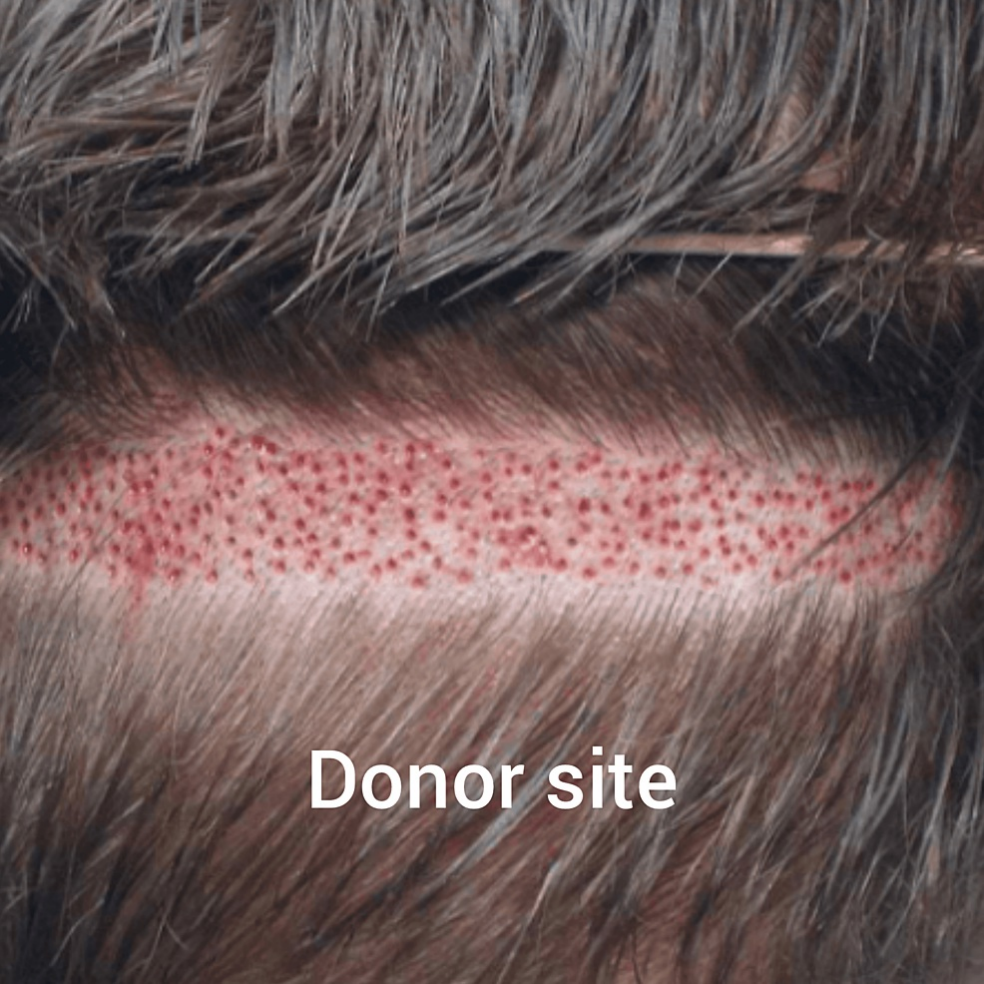
Newer technique of donor site harvest attempting to reduce the posterior scalp scar The image has been reprinted (adapted) with permission from iStockphoto (2023)

This technique is not without its limitations. Enough donor hairs must be available to cover the area of hair loss. The patient is generally left with scars at the donor site, a significant drawback of this technique (Figure 4).

This technique has led the scientific community in search of more novel approaches, namely, stem cell applications to prevent the need for donor sites.

**Figure 5:**
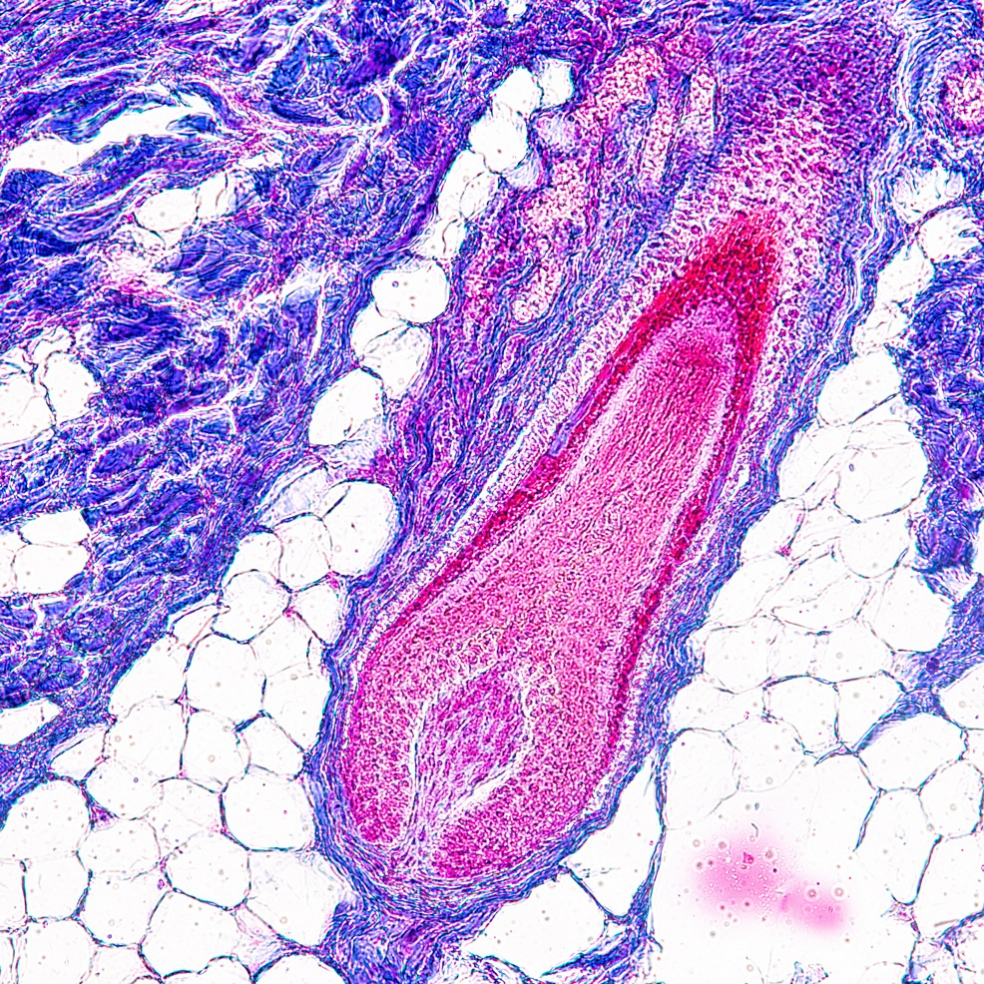
Histologic view of hair follicle The image has been reprinted (adapted) with permission from iStockphoto (2023)

In order to understand stem cell potential, it is important to understand human hair growth cycle. There are three phases in a growth cycle. Anagen is the growth phase in which the hair will grow up to 1 cm per month. This may last up to seven years. In catagen, a two-week transitional phase occurs when the hair shaft separates from the follicle and enters the inactive telogen phase. Telogen phase may last up to four months before hair starts to grow lasting 2-7 years. Subsequently, hair starts growing from its follicle (Figure 5).

It is believed that in hair loss, the hair progenitor cells are reduced; however, the hair follicle stem cells are still viable and present. Therefore, the reactivation of these hair follicle stem cells back to the anagen phase could reverse and regenerate the lost hair [16]. The hair growth relies on several factors in order to continue its maturation. These factors include proteins to create hair itself, blood supply to bring nutrients to the follicle, oil production to keep hair shiny and prevent brittleness, and growth factors to stimulate hair growth and keep follicles productive and active. The deficiency in any of these factors could lead to the loss of hair and alopecia [17].

Currently, there are over 4700 National Institutes of Health (NIH)-sponsored clinical trials underway to understand all the pathways and possible application of stem cell therapy [18]. There are two types of stem cell sources, namely, embryonic stem cell from the umbilical cord and mesenchymal stem cells from other sources such as bone marrow, adipose tissue, or the hair follicle itself. Studies have looked at each component of these essential factors in the search for more effective treatment for alopecia. One factor that has been explored is the importance of chemical signals in follicular physiology. Researchers at the Rockefeller University looked at the interplay of chemical signals such as Wnt and Noggin [19]. These signals are some examples of molecules that influence stem cells to begin differentiation into hair follicles. If no signals are sent to the follicle, then the follicular degeneration starts, and hair growth stops. Some investigations are looking at the signals involved in this process and how to manipulate the signal system in their favor. University of California, Los Angeles (UCLA) investigators [20] have discovered that two drugs, RCGD423 and UK5099, can affect and activate mesenchymal stem cells harvested from hair follicles using different pathways, which transmits information from outside the cell to the nucleus of the cell. RCGD423 leads to the activation of the cell signaling pathway Janus kinase (JAK)-signal transducer and activator of transcription (STAT). JAK-STAT activation leads to the increased production of lactate, and this in turn drives hair follicle stem cell activation and quicker hair growth. UK5099 blocks the pyruvate entering mitochondria of a cell leading to lactate production. This lactate production has been shown to increase the activity of follicle and hair cell growth.

In other studies, attempts have been made to inject stem cell directly into the scalp for growth. Gentile et al. [21] biopsied the bulge of hair follicle. The punch biopsies are trimmed to 2×2 mm width, and fat is removed. The remaining tissue is placed in 1.2 ml of normal saline and centrifuged to separate the cells. The resulting suspension and cells are injected to a depth of 5 mm into planned site. The hair density was checked compared to controls, and it was noticed that there was an improvement in the density of hair in AA patients. Studies such as these show the great potential that stem cells may have in the development of targeted treatments. Other studies have used mesenchymal cells as a source of stem cells. Hardy [22] describes how embryonic stem cells and adult mesenchymal cells can be used to generate new hair or assist with new growth in adults. The abilities and availability of mesenchymal stem cells in hair follicles make it a potential donor site for the harvesting of stem cells to be used in the replacement or regeneration of lost hair.

Some contemporary studies are also looking at how stem cells can replace dead follicles of the scalp and the application of growth factors and molecules that reinitiate previously inactive follicles. Li et al. [23] looked at stem cells not as a replacement but as an adjunct to existing hair follicles in the treatment of AA. AA is the most common autoimmune disease that affects hair follicles leading to hair loss. In this study, investigators provided “stem cell educator therapy,” a process in which a patient’s autologous blood is circulated through a closed loop system with multipotent stem cells (cord blood stem cells, CB-SC) and returned back to the patient after the “education” process. It was observed that this therapy led to the formation of a ring of transforming growth factor-beta (TGF-β) around the hair follicle that provided protection of the follicle from the immune system. This demonstrates another potential application of stem cells in hair growth regulation in alopecia.

Lei et al. [24] observed the key molecules in multistage morphogenesis of tissues. The activation and inhibition of these molecules provide great insight into how stem cells are programmed to create specific three-dimensional tissue structures. In this instance, adult skin cells and stem cells appear to have potential for use in regenerative medicine, including that of hair follicles. An understanding of how cells interact together to create a three-dimensional structure is essential to the successful development of the next stem cell research.

Gentile et al. [25] looked at the isolation of autologous mature stem cells from patients’ own biopsy specimen and injected it in the scalp of 11 patients. They showed that there was an increase in hair density in 29% of the patients receiving the injection. This suggests that stem cells do have potential to be used as an injection in improving hair growth, but further study is being done to better understand if this is secondary to the stimulation of inactive existing stem cells or new cell growth regeneration. In comparison, studies show a 0.4%-2% increase in density in 5% minoxidil-treated patients [26]. Modality such as platelet-rich plasma (PRP) injection has been shown to increase hair density by approximately 29% [27]. Al-Ghadban et al. [28] demonstrated that adipose tissue has a potential to convert to adipose-derived regenerative cells. Their studies demonstrated that injecting adipose-derived regenerative cells next to regressed follicle increase the growth and density of the hair over a three- and six-month period. The other areas of investigation focus on the bioactive molecules present in stem cells. Growth factors, chemokines, cytokines, and important regulatory proteins are also contributing to hair growth. The discovery and use of these factors can also help reverse the regression of hair follicle. One particular molecule is the paracrine factor present in the signaling mechanism of the stems cells [29]. The use of rich mediums placed at the location of the follicles can signal the growth and reverse the dormant inactivity of the follicle.

New studies are emerging on the signaling mechanism of hair growth. It is understood that hair follicle stem cells are controlled by the dermal papilla, namely, fibroblasts. The activation of Hedgehog signaling murine fibroblasts increases the heterogeneity of fibroblasts into Wnt5a state. This will activate *Gli1*, *Alx3*, *Sox18*, *Zfp239*, and *Ebf1* genes. This in turn upregulates the production of signal peptide complement C1r/C1s, Uegf, and Bmp1 (CUB)-epidermal growth factor (EGF)-like domain-containing protein 3 (SCUBE3) in fibroblasts of dermal papillae, which in turn activates the follicle, and hair growth resumes [30]. Finally, a new group of aging studies has also shown that aging can be associated with stem cell exhaustion. NIH researchers have shown that with aging, stem cells lose adhesions and start moving away from base of the follicle [31,32]. These studies were part of the overall aging process studies, and hair follicles were a good model for these studies. These researchers have noticed that genes such as *FOXC1* and *NFATC1* in mice are regulators of adhesion molecules, and their activity affects the migration of the stem cells below the skin papillae. The control of this migration may help control aging damage and hair loss.

Stem cell therapy for hair loss also has many limitations. The studies have not been standardized in the preparation of the cells and the quantity used. There has been no study in the time interval between treatments and whether the stem cell injection is permanent. The advantage of one type of donor stem cell such as adipose tissue versus others such as bone marrow or hair follicle has not been studied. There is no standards in extraction method, and finally, there is no great treatment available for massive hair loss. All these issues need to be addressed in future studies.

## Conclusions

Scientists have developed novel approaches to try to use stem cells to improve the result of baldness. The studies described above show promise that stem cells have the potential to enhance medical treatment options currently available for alopecia. Although these studies are in their infancy, there are over 4700 NIH-sponsored clinical trials underway to understand all the pathways and possible application of stem cell therapy. The combination of old techniques and stem cell could eventually allow physicians to better treat disorders such as male pattern baldness or alopecia areata. With further development of this treatment, feelings of confidence among the millions of balding males and females in the United States of America could be restored if the effects are to be permanent.
